# Low levels of HTLV-2 Tax conjugation to ubiquitin and SUMO do not impede Tax-mediated activation of NF-κB

**DOI:** 10.1186/1742-4690-11-S1-O42

**Published:** 2014-01-07

**Authors:** Chloé Journo, Amandine Bonnet, Arnaud Favre-Bonvin, Jocelyn Turpin, Jennifer Vinera, Emilie Côté, Sébastien A Chevalier, Youmna Kfoury, Ali Bazarbachi, Claudine Pique, Renaud Mahieux

**Affiliations:** 1 Oncogenèse Rétrovirale, équipe labellisée Ligue nationale contre le cancer, CIRI, INSERM U1111-CNRS UMR5308, Université Lyon 1, Ecole Normale Supérieure de Lyon, LabEx ECOFECT, Lyon, Cedex 07, France; 2 Institut Cochin, INSERM U1016-CNRS UMR8104, Université Paris Descartes, Paris, France; 3 Department of Internal Medicine, Faculty of Medicine, American University of Beirut, Beirut, Lebanon

## 

Constitutive activation of NF-**κ**B by HTLV-1 Tax is a key event in the process of T lymphocyte immortalization and leukemogenesis. Tax1 is post-translationally modified by both ubiquitin and SUMO. These modifications were originally thought to be required for Tax1-mediated NF-**κ**B activation by allowing recruitment of IKK-γ/NEMO together with Tax1 in Golgi-associated cytoplasmic domains, followed by activation of the IKK complex and RelA/p65 nuclear translocation. Although HTLV-2 Tax also activates the canonical NF-**κ**B pathway, the requirement of post-translational modifications had not been investigated so far. We therefore compared the ubiquitination, SUMOylation, and acetylation patterns of Tax2 and Tax1. We first show that in contrast to Tax1, Tax2 conjugation to endogenous ubiquitin and SUMO is barely detectable while both Tax2 and Tax1 are acetylated. Consistent with these observations, overexpression of the E2 ubiquitin-conjugating enzyme Ubc13 does not affect Tax2 conjugation to ubiquitin and Tax2-mediated NF-**κ**B activation. We further identify the domains surrounding Tax1 lysine residues K4-10 and K6-10 as critical determinants of Tax conjugation to ubiquitin and SUMO, respectively. We finally demonstrate that a non-ubiquitinable, non-SUMOylable, and non-acetylable Tax2 mutant retains a significant ability to activate transcription from a NF-**κ**B-dependent promoter after partial activation of the IKK complex and induction of RelA/p65 nuclear translocation. Taken together, these results thus indicate that Tax2 does not share Tax1 requirements toward ubiquitination and SUMOylation for efficient NF-**κ**B activation and highlight key distinctions between both viral proteins. These results will be discussed in the light of the recent findings from other labs.

